# Investigation of fatal chickenpox outbreak among adults in Northern Bangladesh, 2023

**DOI:** 10.1016/j.ijregi.2026.100903

**Published:** 2026-04-17

**Authors:** Immamul Muntasir, Kyaw Thowai Prue Prince, A.K.M. Dawlat Khan, Mohammad Rashedul Hassan, Md. Omar Qayum, Manjur Hossain Khan Jony, Mahbubur Rahman, Ahmed Nawsher Alam, Tahmina Shirin

**Affiliations:** Institute of Epidemiology, Disease Control & Research (IEDCR), Mohakhali, Dhaka, Bangladesh

**Keywords:** Disease outbreaks, Varicella-zoster virus, Adult mortality, Traditional medicine, Health-seeking behavior

## Abstract

**Objectives:**

Although chickenpox is usually mild and self-limiting, severe complications and death may occur among adults. In April 2023, two adult deaths associated with clinically diagnosed chickenpox in a remote village of northern Bangladesh triggered notification of a suspected outbreak and prompted an investigation to describe its epidemiologic characteristics and contextual factors associated with severe adult cases.

**Methods:**

A mixed-method outbreak investigation was conducted from April 15-19, 2023 in Thakurgaon district. Active case finding, household visits, line listing, and verbal autopsies were performed. Vesicular swabs were tested for varicella-zoster virus (VZV) by polymerase chain reaction (PCR). Environmental assessments and a focus group discussion explored contextual factors, community perceptions, and care-seeking behavior.

**Results:**

A total of 23 confirmed cases were identified, including seven laboratory-confirmed cases. The attack rate was 1.2% and the case fatality rate 8.7%. Adults over the age of 20 years comprised 52.2% of cases and males 60.9%. Both fatal cases were adult males with comorbidities, heavy smoking history, delayed care-seeking, pneumonia, and thrombocytopenia. All samples were PCR-positive for VZV. The epidemic curve suggested propagated transmission. Qualitative findings indicated reliance on traditional healers and delayed formal care.

**Conclusion:**

Chickenpox can result in severe and fatal outcomes among adults in resource-limited settings. Early recognition and timely health care access are critical to reducing mortality.

## Highlights

• Adult predominance observed in a rural chickenpox outbreak in Bangladesh.
• Fatal adult cases linked to delayed care-seeking and comorbidities.
• Thrombocytopenia and pneumonia identified in both fatal cases.
• Strong reliance on traditional healers delayed formal health care access.

## Introduction

Chickenpox or primary varicella is an infectious disease caused by the varicella-zoster virus (VZV). The incubation period ranges from 10 to 21 days. The illness is characterized by a low-grade fever, malaise, and a widespread, itchy rash that consists of vesicles. Before the onset of a rash, there may be mild prodrome and adults may experience fever and malaise for 1-2 days. It is generally a self-remitting disease. However, adults and immunocompromised individuals may experience more severe outcome and a higher risk of complications, such as bacterial skin infections, encephalitis, and pneumonia. Patients are infectious from 1 to 2 days before the onset of the rash until all lesions have crusted over [[1], [2], [3]].

In countries with temperate climates, most individuals develop immunity to VZV by adolescence. In contrast, serological studies indicate that in tropical countries infection often occurs later in life, during late adolescence or adulthood, resulting in a higher proportion of adult cases and increased risk of complications and mortality [4,5].

Bangladesh has a subtropical climate in the central-north region and a tropical climate in the south. In Bangladesh, chickenpox cases follow a seasonal pattern, with increased incidence typically observed between mid-February and mid-April, during the spring season [6]. Although chickenpox occurs annually in Bangladesh, its epidemiology remains poorly documented, with limited information on outbreak characteristics, severity, and outcomes.

In late March 2023, two patients with clinically diagnosed chickenpox in a village of Thakurgaon, a northern district of Bangladesh, were hospitalized and subsequently died from complications. This event raised public health concern in the locality. Therefore, the Civil Surgeon (district health manager) of Thakurgaon notified the Institute of Epidemiology, Disease Control and Research (IEDCR), Dhaka. Consequently, a National Rapid Response Team was mobilized by IEDCR to investigate the epidemiologic characteristics and contextual factors associated with these fatal cases and contain the outbreak.

## Methods

**Figure 1:**
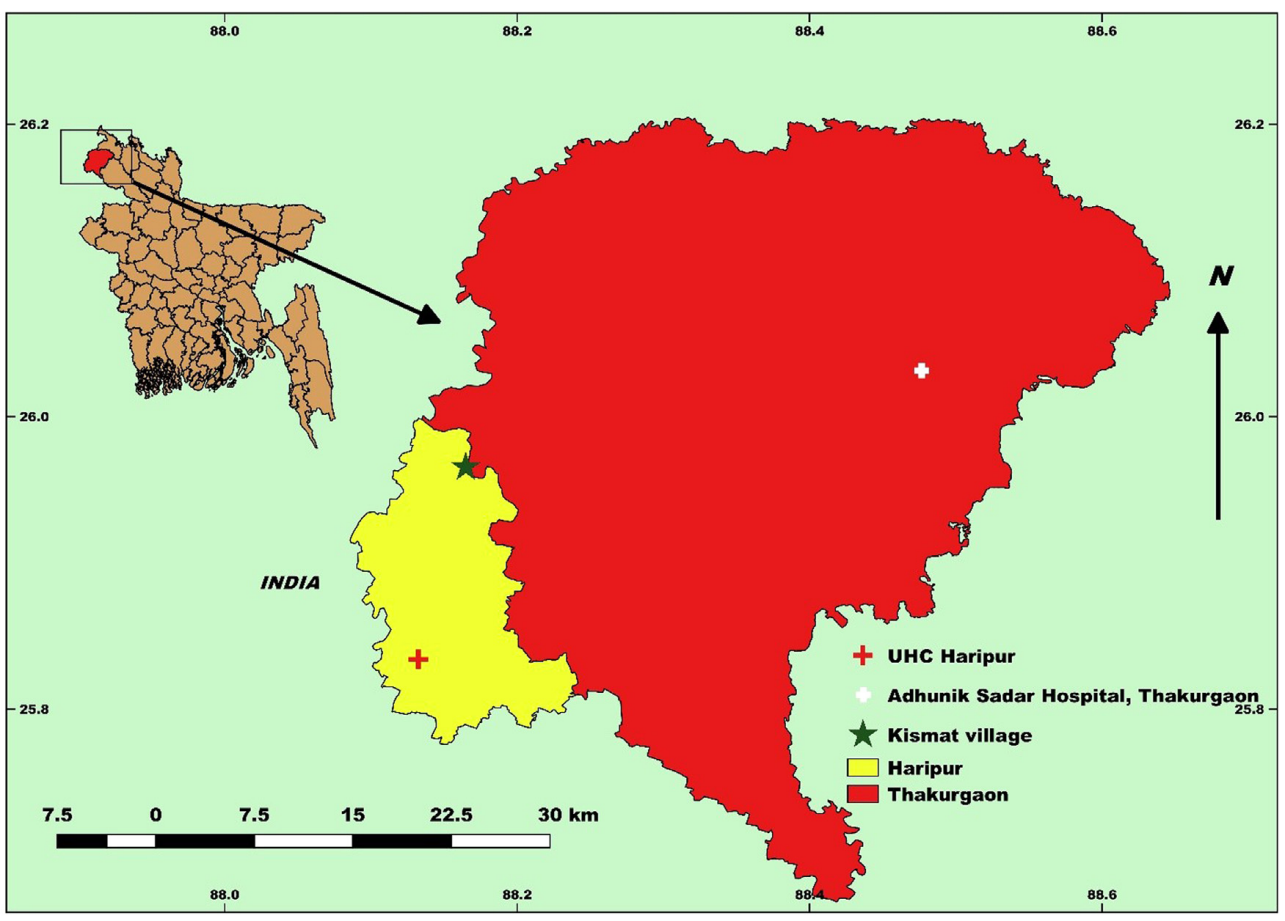
Map of places of investigation of chickenpox outbreak at Thakurgaon, Bangladesh, 2023.

The outbreak investigation was conducted from April 15-19, 2023 comprising of epidemiologic, environmental, laboratory and anthropological studies. The investigation took place in Kismat village, under Haripur sub-district of Thakurgaon district, the Upazila Health Complex (UHC) in Haripur, and Adhunik Sadar Hospital in Thakurgaon (Figure 1).

### Epidemiologic investigation

For the epidemiologic investigation, we constructed a case definition based on the Centers for Disease Control and Prevention (US CDC) guidelines for varicella outbreaks [7]. The clinical case definition for varicella was an illness characterized by the acute onset of a generalized maculopapular vesicular rash without any other apparent cause. In vaccinated individuals who developed varicella more than 42 days post-vaccination, the rash could appear atypical, presenting as maculopapular with few or no vesicles.

We classified cases as probable or confirmed. A probable case was defined as any person from Kismat village who met the clinical case definition but was neither laboratory-confirmed nor epidemiologically linked to a confirmed case. A confirmed case was either laboratory-confirmed or met the clinical case definition and was epidemiologically linked to a confirmed case.

We conducted verbal autopsies for the two deceased cases and performed active case finding through household visits and interviews with community members in Kismat village. A case record form was developed to collect clinical features and exposure histories from all case patients. We conducted an active community-based search for additional probable cases through door-to-door visits to households in the affected area. During these visits, household members were interviewed using a structured questionnaire to identify individuals meeting the case definition. In addition, we reviewed records at the UHC and Sadar Hospital to identify other probable cases who may have sought care. We then prepared a line list to gather information on all case patients, which helped us understand the disease’s extent.

### Laboratory investigation

We collected vesicular swab specimens from probable cases with active vesicular lesions. Samples were transported under cold chain to the IEDCR and tested for VZV using polymerase chain reaction at the virology laboratory.

### Anthropological investigation

The investigating team visited the households of the cases and conducted informal discussions with them, their family members, and community residents living in close proximity to the affected households. These discussions aimed to explore community knowledge of chickenpox and gather information related to the potential source and spread of the outbreak.

In addition, the team conducted a focus group discussion (FGD) with family members of the cases to understand local knowledge and health-seeking practices, including treatment options, barriers to accessing modern health care, isolation, and preventive measures related to chickenpox. The FGD was voice-recorded.

### Data analysis

Data analysis included a descriptive analysis of the outbreak by time, place, and person. Categorical variables were summarized as counts and percentages, whereas continuous variables were described using measures of central tendency and dispersion. We calculated the overall attack rate and case fatality rate. The overall attack rate was calculated using the village population (N = 1950), obtained from local administrative records and cross-checked during field visits, whereas the case fatality rate was calculated using only the number of identified confirmed cases as the denominator. We constructed an epidemic curve to illustrate the magnitude and progression of the outbreak. Data analysis was conducted using Stata version 17.0.

The audio recordings were transcribed verbatim. The team collected and compiled all the field notes from informal discussions. The team followed thematic analysis and read the interview transcripts several times to identify emerging concepts and themes [8]. Codes were developed inductively and grouped into broader thematic categories. Data from informal discussions were analyzed similarly and then compared with themes identified from the interviews and FGDs to assess consistency and identify convergent and divergent findings. This triangulation across data sources enhanced the rigor and credibility of the study findings [9].

## Results

### Descriptive epidemiology

A total of 23 confirmed chickenpox cases were identified, including seven cases with active disease at the time of the investigation; all seven were laboratory-confirmed. The remaining cases met the clinical case definition and were epidemiologically linked. The overall attack rate was 1.2% (23 of 1950) and the case fatality rate was 8.7% (two of 23). Two (8.7%) cases required hospitalization; both resulted in death. Among the cases, 39.1% (nine of 23) were female and 60.9% (14 of 23) were male (Table 1). The mean age of cases was 23 years (SD ± 13.8 years). The ages of the cases ranged from <1 to 53 years. The most affected age group was above 20 years, with 52.2% (12 of 23) (Table 1).

**Table 1:** Gender and age group distribution of chickenpox cases in Kismat village, Haripur, Thakurgaon, 2023 (N = 23).

Demographic	Category	Frequency	Percentage (%)
Gender	Female	9	39.13
	Male	14	60.87
Age group (year)	<1	1	4.35
	1-10	4	17.39
	11-20	6	26.09
	21-30	5	21.73
	31-40	4	17.39
	41-50	2	8.70
	51-60	1	4.35

**Figure 2:**
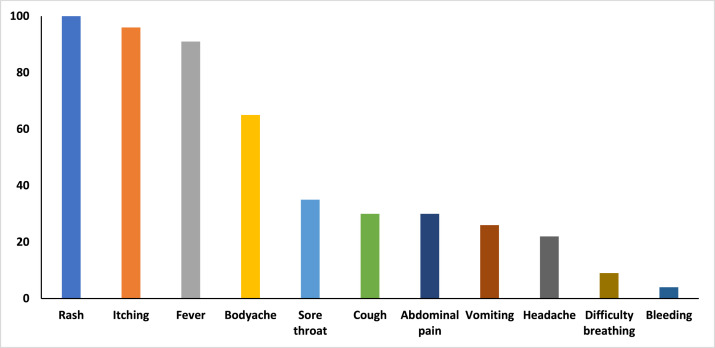
Symptoms reported by chickenpox cases in Kismat village, Haripur, Thakurgaon-2023 (N = 23).

**Figure 3:**
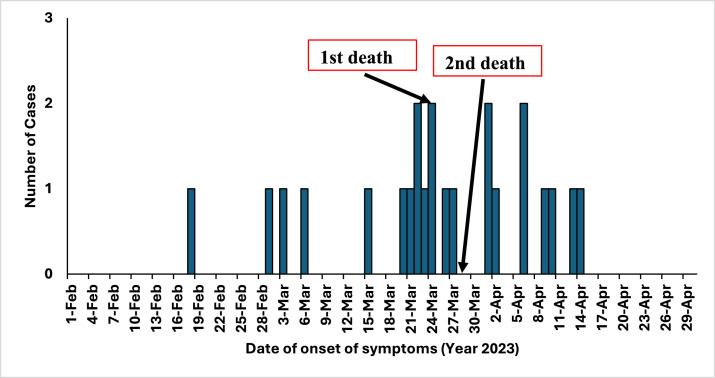
Epicurve of chickenpox outbreak at Kismat village of Haripur upazila, Thakurgaon, 2023 (N = 23).

The most common symptoms experienced by the cases were rash (100%), itching (96%), fever (91%), body ache (65%), and vomiting (34%) (Figure 2). The epidemic curve is suggestive of a propagated source outbreak, indicating person-to-person transmission. The date of onset of symptoms of the first detected case was February 17, 2023. No new cases were reported after April 14, 2023 (Figure 3).

### Earliest identified case

The earliest identified case was a 53-year-old male hailing from Kismat village. The village is approximately 15 km from the UHC of Haripur. The case was a farmer who frequently visited other places outside the village for work. His rash first appeared on February 17, 2023 on the chest and then the back, followed by the limbs and face. He did not seek any treatment from the nearest health center. His vaccination status was unknown. According to him, the duration of rash was around 9-10 days. Along with rashes, he experienced intermittent fever and itching all over the body. He did not have any other diseases and was not on any immunosuppressive drugs. He did not recall any potential exposure to chickenpox infection in the weeks before the onset of rash because he frequently visits different places for his work. The second reported case in the village was also among his family.

### Verbal autopsy

From the verbal autopsy of two deceased individuals, we found that the first deceased individual (index case) was a 35-year-old male resident of Kismat village with a history of heavy smoking for 10 years and had chronic cough. On March 21, 2023, he developed sudden abdominal pain with distension, followed by fever, with maculopapular vesicular rash that initially appeared in the trunk and subsequently spread to the limbs. He initially consulted a doctor and received treatment for dyspepsia and suspected chickenpox. His condition worsened, and he was admitted to Thakurgaon Sadar Hospital on March 23, 2023. However, his condition deteriorated further, and he developed vomiting with blood and reddish urine. Complete blood count report indicated thrombocytopenia. Despite treatment, he died on March 24, 2023. The cause of death was determined by the treating physician to be irreversible cardiovascular failure, with community-acquired pneumonia and chickenpox.

The second deceased individual was a 45-year-old male resident of Kismat village who passed away on March 27, 2023. He had a history of heavy smoking for 20 years and had chronic cough and diabetes. On March 20, 2023, he developed body aches and malaise, followed by a fever with a maculopapular vesicular rash that spread across his body in a centripetal pattern. Despite visiting a homeopathic doctor and a traditional healer and receiving treatment from both, his condition continued to deteriorate, and he developed chest pain and breathlessness. He was subsequently taken to Dinajpur Medical College & Hospital on March 26, 2023 but was later referred and admitted the same day to the intensive care unit of Zia Heart Foundation. Unfortunately, his condition continued to worsen, and he passed away the next morning. Blood report indicated thrombocytopenia and hyponatremia. Hospital documentation indicated irreversible cardiovascular failure with pneumonia in a patient with clinically diagnosed chickenpox and diabetes mellitus.

### Laboratory findings

All seven vesicular swab samples taken from the active cases during the field investigation were positive for VZV in the virology laboratory of IEDCR.

### Anthropological findings

#### Knowledge and belief on chickenpox among the community

The anthropological findings from informal discussions and FGD indicated that community members believed that chickenpox is an illness that occurs due to increased temperature after winter. They locally referred to this illness as “*kalche*” (blackish spot), or “*ghuta*”, or “*basanta.*” The community members also believed that this disease is attributable to sick animals, particularly, poultry. In addition, they reported person-to-person transmission of chickenpox disease through close proximity and touching the patient with chickenpox.

Furthermore, the community members reported that every year this illness occurred in limited numbers and particularly among children. However, the community members were concerned because this outbreak happened among large number of adults with fatalities.

A 60-year-old male respondent said: “Last 40-50 years, I don’t remember any deaths and large number of patients in our village. We usually observed chickenpox among children; this year, adult male are prone.”

#### Health-seeking practice for chickenpox among the community

The community members reported traditional practices for treating chickenpox in their community. They reported bathing patients with chickenpox with boiled water with neem (Indian lilac) leaves and used neem leaves on the patient’s bed.

A 45-year-old female respondent said: “We used neem water and neem leaves on the bed to quick recovery and prevent transmission of chickenpox to other people.”

The community members also reported that some households arranged separate beds for affected individuals, particularly, among those who could afford, to prevent person-to-person transmission. However, most community members were reluctant to isolate their loved ones, despite the risk of infection. In addition, some community members reported applying ashes derived from burned cow dung to the lesions to relieve itching and discomfort.

The community members also reported that they avoid beef, fish, cow milk, and bathing until the chickenpox lesions had dried. They believed that these foods can increase the number and size of lesions and scars due to itching. The community members sought treatment from traditional healers locally called “*Kaviraj*” or “*Hujur*”. The community members reported that when someone developed chickenpox, they went taking water, oil, or ghee to the local traditional healer, and the traditional healer recited their spiritual verses over these items and advising them to apply on the patient’s body. The traditional healer also sometimes prescribed taking sweets to the patients.

A 50-year-old female respondent said: “We feed sweet to our patients, so that if any supernatural or evil force possess into the patient’s body, they will leave after taking the sweets.”

Furthermore, the community members reported that some cases family took homeopathic medicine. The community members also reported that when a patient’s condition becomes serious and deteriorates, they seek modern treatment, particularly, admission into the hospital.

A 40-year-old male respondent said, “Previously, no death was occurred due to chickenpox in our village. Two patients were very severe and we admit them to hospitals but they died.”

The community members also opined that traditional treatments are easily available and cost-effective compared with modern treatment so they prefer to seek traditional treatment.

### Public health actions taken

Local health authorities initiated public health actions before the arrival of the outbreak investigation team, and these activities continued concurrently with the field investigation. The investigation team worked closely with these authorities to alert health care providers and strengthen reporting of suspected chickenpox cases. We advised infected individuals to maintain isolation and avoid close contact with high-risk groups, including pregnant women, infants, and immunocompromised persons. During the field investigation, we provided health education to community members through household visits, FGDs, and engagement with local health care providers. We highlighted the contagious nature of chickenpox, early recognition of symptoms, warning signs of severe disease, and the importance of timely care-seeking. We also emphasized identifying individuals at higher risk of severe chickenpox, including adults without a history of infection or vaccination, to encourage early presentation to health care facilities and appropriate clinical management. To monitor ongoing transmission, we supported active surveillance for additional cases for two incubation periods (42 days) after rash onset in the last identified case until May 26, 2023 to ensure interruption of transmission and confirm the end of the outbreak.

## Discussion

This field investigation documented a chickenpox outbreak in a remote rural area of a northern district of Bangladesh. This investigation highlights the need to reconsider chickenpox as a potentially severe adult disease. Although chickenpox is generally considered a mild, self-limiting illness that typically confers long-term immunity after infection, this particular event was notable for the predominance of adult cases and the occurrence of two deaths. Such findings are consistent with evidence from other tropical countries, where chickenpox infection often occurs later in life and is associated with an increased risk of complications among adults [[10], [11], [12], [13], [14]].

The occurrence of two adult deaths in this outbreak underscores the potential severity of chickenpox among adults, particularly, those with underlying health conditions. Both deceased individuals had chronic comorbidities and one of them sought medical care several days after symptom onset. Previous studies have demonstrated that adult chickenpox infection is associated with a higher risk of severe complications, including pneumonia and multi-organ involvement, compared with childhood infection [1]. Also, the history of heavy smoking in both fatal cases may have contributed to disease severity, particularly, through increased vulnerability to respiratory complications [15]. Delayed care-seeking and limited access to timely medical management may have contributed to the adverse outcomes observed in this outbreak [2].

The outbreak occurred during the spring season, which corresponds to the known seasonal pattern of chickenpox in Bangladesh and other tropical settings. Previous studies from Bangladesh and neighboring regions have similarly reported increased chickenpox incidence during this period [6,[10], [11], [12], [13], [14]]. Although climatic factors were not systematically assessed in this investigation, the timing of the outbreak aligns with earlier observations of seasonal clustering of varicella cases in spring months [6].

Qualitative findings indicated that community members were aware of the contagious nature of chickenpox but frequently preferred traditional treatment methods over formal health care. This preference was shaped by long-standing cultural practices, perceived effectiveness of traditional remedies, ease of access, and previous experiences of recovery without medical intervention. Similar reliance on traditional or non-allopathic care has been documented in other outbreak investigations and should be interpreted within the context of limited access to healthcare services and the typically self-limiting course of chickenpox [16].

In addition, both deceased patients had documented thrombocytopenia, which has been reported as an uncommon but clinically significant complication of varicella infection, particularly, among adults. Varicella-associated thrombocytopenia is thought to occur through immune-mediated platelet destruction, bone marrow suppression, or severe systemic inflammatory response and has been linked to hemorrhagic manifestations and poorer clinical outcomes [16,17]. The presence of thrombocytopenia in both fatal cases in this outbreak suggests a possible marker of severe disease and may have contributed to the rapid clinical deterioration observed. Early recognition of hematologic abnormalities, including thrombocytopenia, may, therefore, be critical for identifying adult patients with chickenpox at higher risk of complications and guiding timely referral and intensive clinical management.

Importantly, underreporting of chickenpox cases is likely in Bangladesh because many individuals with mild illness do not seek care at health facilities. Consequently, outbreaks may remain undetected unless severe cases or deaths occur. The detection of this outbreak after hospitalization and deaths highlights gaps in routine surveillance for chickenpox and underscores the need for improved community awareness, early case detection, and timely reporting [6]. In addition, VZV vaccination is not currently included in the routine childhood immunization program in Bangladesh, which may contribute to the continued susceptibility of the population and the occurrence of outbreaks. Consideration of vaccination strategies, particularly, for high-risk groups, could be explored as part of long-term prevention efforts [18].

Overall, this outbreak highlights the importance of recognizing chickenpox as a potentially severe disease among adults in tropical, resource-limited settings. Strengthening community awareness regarding early symptoms and potential complications, improving access to timely health care, and sensitizing health care providers to the management of adult chickenpox may help prevent severe outcomes in future outbreaks [1,2].

### Limitations

This investigation had several limitations. Underreporting is likely because individuals with mild illness may not have been identified or may not have sought medical care, which could have led to an underestimation of the outbreak magnitude. Information collected through verbal autopsies and interviews was subject to recall bias. In addition, qualitative findings were based on a single FGD with a limited number of community members and may not capture the full range of community perspectives.

## Conclusion

This field investigation documented a chickenpox outbreak in a remote village of Bangladesh that affected multiple age groups, with a predominance of adult cases and two deaths. The findings demonstrate that chickenpox, often regarded as a mild illness, can lead to severe outcomes among adults, particularly, those with underlying health conditions, when access to timely and appropriate medical care is delayed. Community perceptions and reliance on traditional treatment practices were documented and, in some cases, appeared to influence care-seeking behavior. These observations underscore the need to strengthen community awareness of early symptoms, modes of transmission, and warning signs of severe disease that require urgent medical attention, as well as to encourage early care-seeking from formal health care services. Engagement of community leaders and trusted local stakeholders is essential to address cultural barriers and promote timely health care utilization. In parallel, training health care providers on evidence-based clinical management of chickenpox, especially for adults and high-risk individuals, should be prioritized. Finally, targeted prevention strategies for high-risk groups, including consideration of vaccination in line with national policy and resource feasibility, may help reduce morbidity and prevent fatal outcomes during future outbreaks.

## Declaration of competing interest

The authors have no competing interests to declare.
